# Using volunteered geographic information to assess mobility in the early phases of the COVID-19 pandemic: a cross-city time series analysis of 41 cities in 22 countries from March 2nd to 26th 2020

**DOI:** 10.1186/s12992-020-00598-9

**Published:** 2020-09-23

**Authors:** Matia Vannoni, Martin McKee, Jan C. Semenza, Chris Bonell, David Stuckler

**Affiliations:** 1 Department of Political Economy, King’s College London, Bush House North East Wing, 30 Aldwych, London, WC2B 4BG UK; 2 Faculty of Public Health and Policy, London School of Hygiene and Tropical Medicine, 15-17 Tavistock Place, London, WC1H 9SH UK; 3 Dondena Centre for Research on Social Dynamics and Public Policy and Department of Social & Political Sciences, Bocconi University, Milan, Italy; 4 European Centre for Disease Prevention and Control, Gustav III:s Boulevard 40, 169 73 Solna, Sweden

## Abstract

**Objectives:**

Restricting mobility is a central aim for lowering contact rates and preventing COVID-19 transmission. Yet the impact on mobility of different non-pharmaceutical countermeasures in the earlier stages of the pandemic is not well-understood.

**Design:**

Trends were evaluated using Citymapper’s mobility index covering 2nd to 26th March 2020, expressed as percentages of typical usage periods from 0% as the lowest and 100% as normal. China and India were not covered. Multivariate fixed effects models were used to estimate the association of policies restricting movement on mobility before and after their introduction. Policy restrictions were assessed using the Oxford COVID-19 Government Response Stringency Index as well as measures coding the timing and degree of school and workplace closures, transport restrictions, and cancellation of mass gatherings.

**Setting:**

41 cities worldwide.

**Main outcome measures:**

Citymapper’s mobility index.

**Results:**

Mobility declined in all major cities throughout March. Larger declines were seen in European than Asian cities. The COVID-19 Government Response Stringency Index was strongly associated with declines in mobility (*r =* − 0.75, *p* < 0.001). After adjusting for time-trends, we observed that implementing non-pharmaceutical countermeasures was associated with a decline of mobility of 10.0% for school closures (95% CI: 4.36 to 15.7%), 15.0% for workplace closures (95% CI: 10.2 to 19.8%), 7.09% for cancelling public events (95% CI: 1.98 to 12.2%), 18.0% for closing public transport (95% CI: 6.74 to 29.2%), 13.3% for restricting internal movements (95% CI: 8.85 to 17.8%) and 5.30% for international travel controls (95% CI: 1.69 to 8.90). In contrast, as expected, there was no association between population mobility changes and fiscal or monetary measures or emergency healthcare investment.

**Conclusions:**

Understanding the effect of public policy on mobility in the early stages is crucial to slowing and reducing COVID-19 transmission. By using Citymapper’s mobility index, this work provides the first evidence about trends in mobility and the impacts of different policy interventions, suggesting that closure of public transport, workplaces and schools are particularly impactful.

## Summary box

### What is already known on this topic?

Governments across the global are experimenting with a range of policy interventions to restrict movement in populations. Yet their impact is not well understood. There is an urgent need to understand how alternative policy approaches to restricting movement can impact on population mobility trends.

### What this study adds.

Our study finds that policy restrictions markedly reduced population-wide mobility. Closing public transport, workplaces and schools have among the largest associations with mobility declines.

## Introduction

Even before the advent of germ theory, authorities employed measures to restrict movement to reduce the spread of disease, exemplified by the introduction of quarantine by the Venetian Republic. The emergence and global spread of COVID-19 has given new prominence and importance to such measures, although in a world that has changed enormously in the past five centuries. On the one hand, the development of motorised transport has vastly increased the opportunities for mobility. On the other, the spread of mobile phones, of which the world’s 7.8 billion people now own an estimated 14 billion [1], has generated a wealth of information about those who are moving. As governments worldwide adopt measures unimaginable even a few months ago to restrict the intermixing of people with the intention to control the pandemic, there is a need to find novel ways to evaluate their effectiveness.

The data collected from mobile phones can include not only whom users call but also users’ location. At first, this was obtained by triangulating data from phone masts, but this has been supplemented by data from the geographical positioning system (GPS) chips included in smartphones [2]. The data collected provide a rich source of information on patterns of travel and are used extensively by software companies, often via apps that users have permitted to track their location, for example so that users of Uber and other taxi companies and their drivers can locate each other, and so that public transport users can use an app to discover when the next bus is due. The volunteered geographic information generated is increasingly used by transport planners to develop routes and timetables [3].

The richness of these data and their ability to track movement in real time have attracted the attention of epidemiologists. For example, combining phone-derived mobility data with information on climatic conditions was shown to improve the ability to predict the spread of dengue in Pakistan [4]. Similarly, mobile phone data improved the ability to model schistosomiasis patterns in Senegal [5] and HIV in Côte d’Ivoire [6]. There are, however, a number of limitations, the most important of which is that data may be unavailable due to concerns about privacy, as was the case in west Africa during the Ebola outbreak [7].

Inevitably, epidemiologists are now looking to this source of data to help understand the dynamics of the COVID-19 virus pandemic and, especially, to evaluate the impact of countermeasures, many of which seek to reduce mixing of people. Thus, researchers in Seattle have used data from Facebook Data for Good, a resource developed for tracking mobility during disasters, to show that restrictions on movement were very effective in reducing journeys into the city [8]. The Swedish not for profit organisation Flowminder has been established to facilitate the availability of mobile phone data for those responding to health crises. However, most of these initiatives are, in practice, limited to ad hoc studies in single locations [9]. While these can answer the important question of whether a particular countermeasure was effective in reducing mobility in a particular place, individually they cannot show what happens in others. While it is, in theory, possible to obtain data from multiple locations, this would involve a major logistic exercise to do so from many different telecommunications companies in countries with different legal frameworks.

While recognising the importance of taking context into account, we sought to investigate whether and which countermeasures have been effective across diverse country settings. We have identified one source of volunteered geographic information collected across many different locations, collated by the developers of the Citymapper app. Citymapper gathers large amounts of open source data generated by transport authorities, local transit authorities and users, and processes it to provide users with information on the easiest and fastest way of getting from one place to another. It is used primarily by those making journeys by public transport, walking, cycling, and taxis, with the areas covered reflecting the availability of open data but, generally, covering entire metropolitan areas. Users enter their starting point (manually or linked to the GPS function in their phone) and destination and the app offers them a choice of routes, including taxis and public transport. Walking instructions are included, using data from OpenStreetMaps. A 2018 report stated that it then had approximately 20 million users but there are no publicly available data on their characteristics [10]. In response to the pandemic, the developers have published data on mobility in cities worldwide, derived from the searches undertaken on the app. Mobility is measured by numbers of journeys planned but CityMapper advise us that a large proportion of planned journeys can be linked to actual journeys made using their GO function and the two are highly correlated, numerically and spatially. It is, as far as we know, the only publicly available data of this type from multiple locations in a large number of countries. Findings from individual cities have been reported in the media [11] but, so far, they have not been related to the timing of implementation of COVID-19 virus countermeasures. We sought to examine the feasibility of using Citymapper data to capture changes in mobility and relate them to the timing of pandemic countermeasures, focusing on the early stages of the pandemic when the greatest variations in policy responses were implemented.

## Methods

**Fig. 1:**
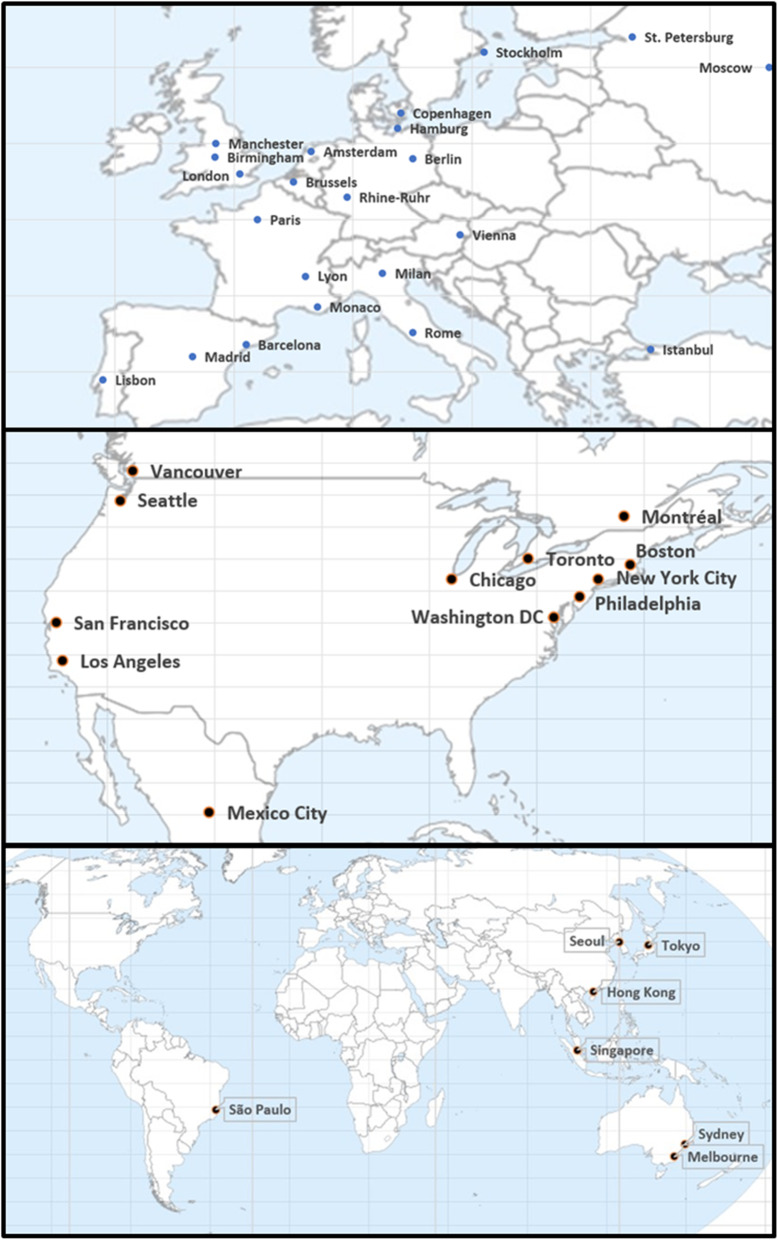
Cities covered by the Citymapper Mobility Index

We extracted mobility from Citymapper’s mobility index for the period 2nd to 26th March 2020 [12]. We included every one of the 41 cities in 22 countries worldwide covered by CityMapper but we note that they do not have any cities in China or India (Fig. 1). CityMapper negotiates individual agreements with authorities in each city to ensure access to data, something that is not possible everywhere and, given the investment required, the company must limit its ambition. The mobility index, published since the onset of the COVID-19 pandemic, reports the volume of trips planned using the app on a particular day compared with a “recent typical usage period”. In most cities this was the 4 weeks between 6th January and 2nd February 2020, but in Paris it was 3rd February-1st March and Hong Kong/Singapore 2nd – 22nd December 2019.Thus, the index can go above 100%, reflecting above average mobility, to below 100%, corresponding to lower than average mobility. Moreover, the sample includes cities that were just starting to experience the first cases, such as Lisbon, as well as cities where a substantial number of cases were already present, such as Milan. Finally, these data represent mobility in the early stage of the pandemic, although this is the period of interest for evaluating the impact of the initial countermeasures.

Data on the social restrictions were taken from the Oxford COVID-19 response dataset [13]. This dataset codes restrictions on movements including school and workplace closures, transport restrictions, and cancellation of mass gatherings, in practically all countries since 1 January 2020. Each intervention is coded as 0 for no measures, 1 for measures that are not legally binding and 2 for those that are mandatory (although coding varies among variables, reflecting the nature of different measures). In addition, the Oxford team has compiled a composite index termed the COVID-19 Government Response Stringency Index. Most of the analyses are undertaken at the level of cities, applying the national data on countermeasures to all cities in the country.

### Statistical modelling

First, we present descriptive time trends for mobility over time. Then we evaluate city-specific slopes (‘fixed effects’ modelling) comparing the changes in mobility before and after the introduction of alternative mobility restrictions. Furthermore, we adjust statistical models for potential secular time-trends, pre-existing mobility rates and other time-invariant factors that could account for between-city differences. Robust standards errors were clustered by city to adjust for spatial correlation within nations. All analyses were conducted using Stata v15.1.

#### Patient and public involvement

There was no patient or public involvement in this study.

#### Data sharing

All data are available from the Citymapper website

#### Transparency statement

The lead author (the manuscript’s guarantor) affirms that the manuscript is an honest, accurate and transparent account of the study being reported, and that no important aspects of the study have been omitted.

#### Role of the finding source

No additional funding was required. Open access fees will be paid by the London School of Hygiene & Tropical Medicine.

#### Dissemination to participants and related patient and public communities

The results will be provided to UK and European COVID-19 bodies that the authors are advising.

## Results

### Trends in mobility during the COVID-19 pandemic

**Fig. 2:**
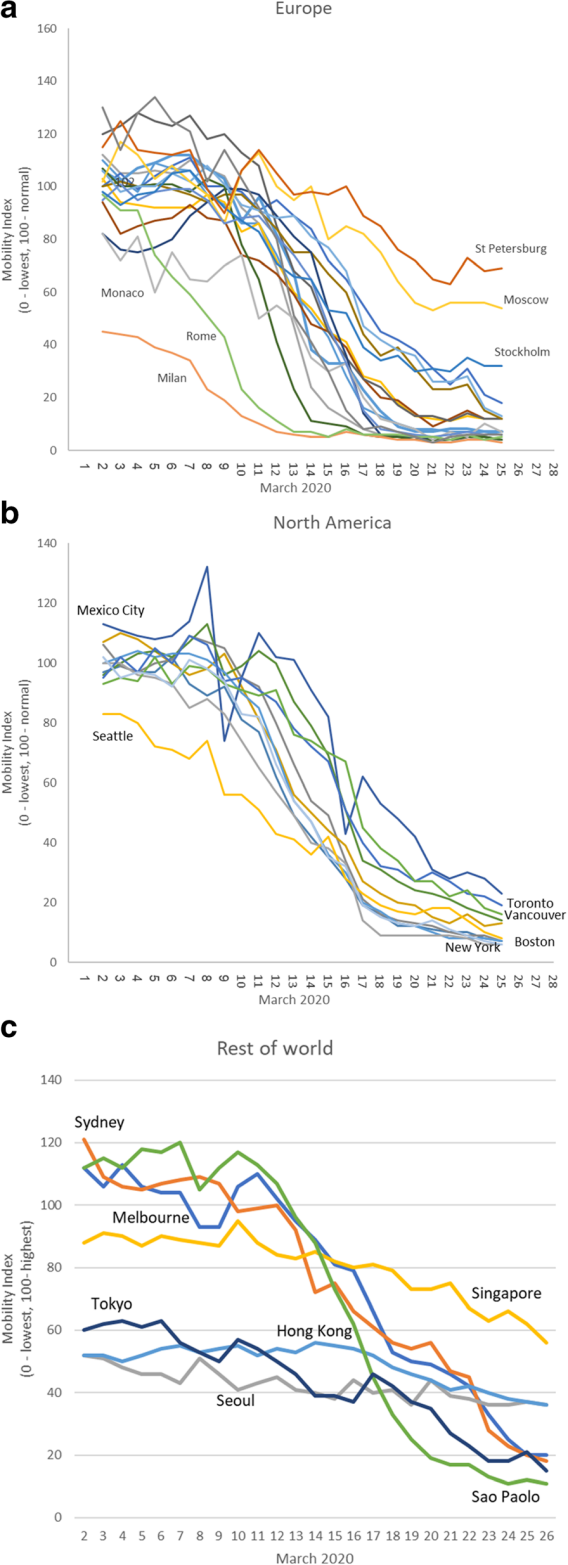
Global Trends in Citymapper Mobility Index, 41 cities

Figure 2 shows trends in mobility from March 2nd to March 26th for cities in Europe, North America and ‘other cities in the Citymapper dataset. In all cities in Europe, there were steep declines, typically in early March, but with some differences. In Milan, mobility was already at under 50% of normal by the start of the period; the earliest clusters of infection had appeared in the Lombardy Region around the 20th February (Additional file 1 Appendix 2). The smallest reductions were in the two Russian cities, which were still above 50% mobility on March 25th, consistent with the slow emergence of COVID-19 in Russia and consequent late adoption of countermeasures. Sweden stands out in western Europe for resisting pressure to take stringent measures to reduce mobility and, other than the Russian cities, it shows the smallest reduction in mobility. In North America, there is a broadly similar trend everywhere, except that Mexico City shows a series of large fluctuations that we are unable to explain and Seattle, where there were a number of early cases, was already experiencing reduced mobility by the time the dataset started. In Asia, where the pandemic started, all cities were already exhibiting reduced mobility by the beginning of the period but further declines were relatively small compared to those seen ‘other cities in the Citymapper dataset. The two Australian cities and Sao Paolo followed similar trajectories to those seen in Europe.

We can see the importance of differential timing of mobility restrictions, including potential anticipatory effects (Fig. 2). Eleven municipalities in the Lombardy region, whose capital is Milan, were placed in lockdown on 21st February. This was extended nationwide on 9th March. Consistent with this, mobility had already fallen in Milan but the reduction in Rome was somewhat later. In contrast, in the United Kingdom, restrictions were introduced nationwide and all three British cities moved in the same way. Notably, in both countries, mobility was falling well before the formal implementation of restrictions.

**Fig. 3:**
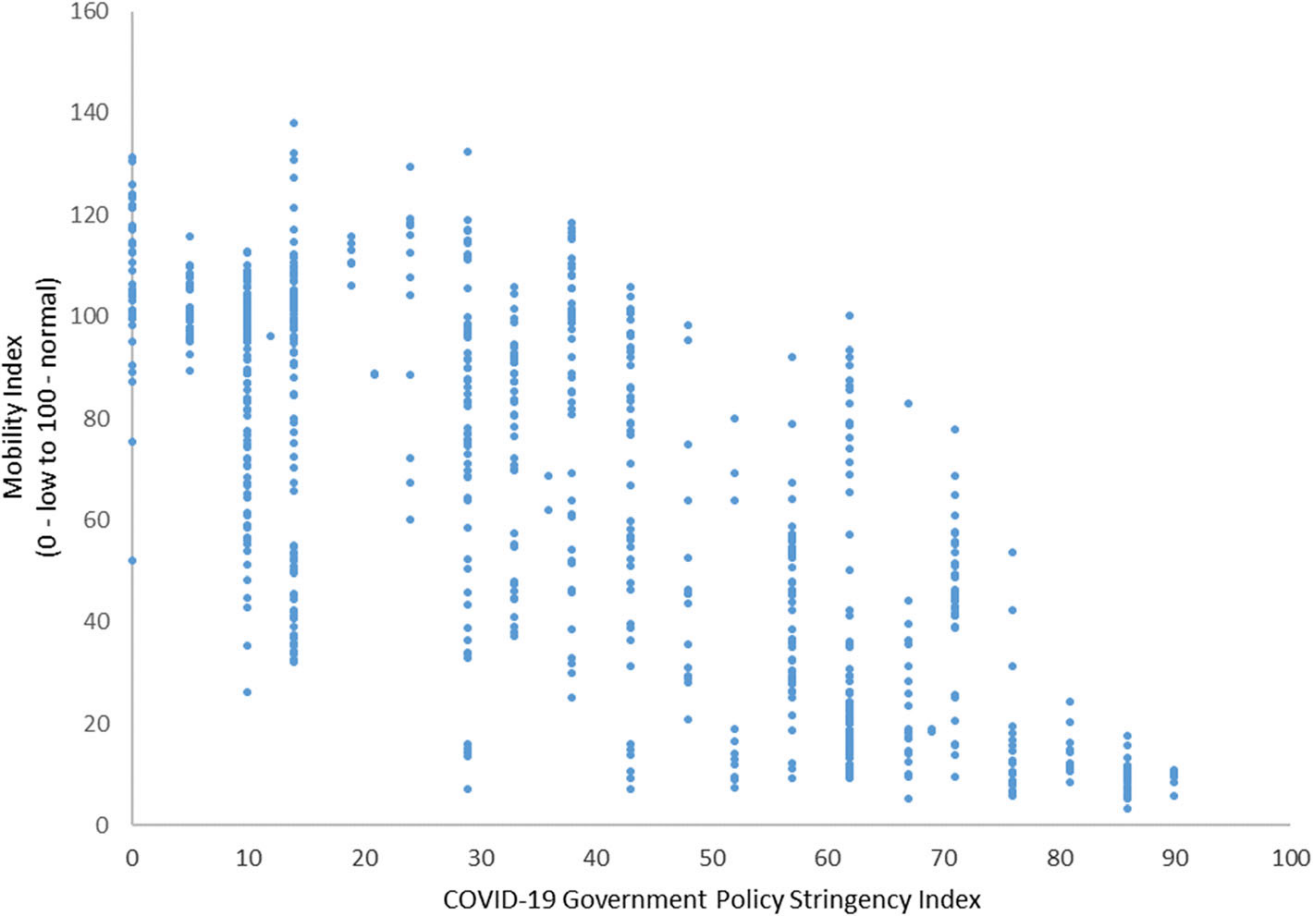
Association of Oxford’s Policy Stringency Index and Citymapper Mobility Index, Pearson’s *r* = − 0.75, *p* < 0.01)

Next, we evaluated the link between stronger government restrictions on movement and the Citymapper Mobility Index. Figure 3 plots changes in mobility in the cities in the sample and the COVID-19 Government Response Stringency Index at country level over time. Every dot is a city-day observation. The figure shows a strong association between the COVID-19 Government Response Stringency Index and declines in mobility (*r =* − 0.75, *p* < 0.001).

### Associations with mobility of alternative policy restrictions on movements

In univariate analyses, all of the measures reported were associated with reduced mobility (see Additional file 1 Appendix 3). However, when including them jointly in the model (Table 1), several were no longer associated and in other cases the magnitude of association diminished slightly (** *p* < 0.01). Clearly, there is a risk of multicollinearity as many measures were implemented simultaneously, but diagnostic tests revealed a variance inflation factor of 1.85, indicating that this was not a major problem.

**Table 1:** Association of policy restrictions with Citymapper’s Mobility Index, 41 cities (fully adjusted model)

	Association with Mobility Index
School closing	−10.0%^**^ [−15.7,-4.36]
Workplace closing	−15.0%^***^ [−19.8,-10.2]
Cancel public events	−7.09%^**^ [− 12.2,-1.98]
Close public transport	−18.0%^**^ [− 29.2,-6.74]
Public information campaigns	−6.68% [− 14.3,0.90]
Restrictions on internal movement	−13.3%^***^ [− 17.8,-8.85]
International travel controls	−5.30%^**^ [− 8.90,-1.69]
Fiscal measures	0.00% [0.00 to 0.00]
Monetary measures	19.8% [−2.61,42.3]
Emergency investment in health care	0.00% [0.00 to 0.00]
Number of City-days	369
R^2^	0.870

Notes: 95% confidence intervals in brackets

^**^ *p* < 0.01, ^***^ *p* < 0.001

After further adjusting for time-trends, we observed that implementing non-pharmaceutical countermeasures was associated with a decline of mobility by 10.0% for school closures (95% CI: 4.36 to 15.7%), 15.0% for workplace closures (95% CI: 10.2 to 19.8%), 7.09% for cancelling public events (95% CI: 1.98 to 12.2%), 18.0% for closing public transport (95% CI: 6.74 to 29.2%), 13.3% for restricting internal movements (95% CI: 8.85 to 17.8%) and 5.30% for international travel controls (95% CI: 1.69 to 8.90). In contrast, as expected, there was no link between fiscal or monetary measures and mobility changes.

### Robustness checks

We performed a series of sensitivity and robustness checks. First, we assessed the external validity of Citymapper data against other data on mobility. In Additional file 1 Appendix 1, we plot the changes in the Citymapper Index in London with changes presented by the British Cabinet Office in journeys in London, collected by the Department of Transport [14], finding that it follows a very similar trajectory (as expected, as Citymapper Index mostly relies on public transportation data). The data for Seattle also closely matched what was reported in the study cited above that used Facebook Data for Good [8], although in that case there were only four data points for comparison. When indexed on 5th March, the first day in common in both datasets, values for three of the four subsequent dates were within one percentage point of each other.

## Discussion

### Principal findings

There is a critical need to understand the impact of early countermeasures intended to restrict mobility during epidemics. Our analyses used one publicly available measure, from Citymapper, to capture changes in mobility in 41 cities worldwide and relate them to the imposition of pandemic countermeasures. Our findings demonstrate that several policy restrictions, notably closures of public transport, workplaces and schools, had a substantial impact on reducing population mobility. Our finding of no such impact of fiscal or monetary measures (which were hypothesised not to have mobility impacts) adds to the specificity and therefore the plausibility of our findings [15].

### Strengths and weaknesses of the study

Our study has several important limitations. Most importantly, the data published by Citymapper are generated from within what is essentially a black box. The company has, however, given us some additional information and, following our request, published some of this on their website. Our robustness checks did, however, show that, at least in two cities, the changes observed correlated with those found using other data sources.

Second, there may be specific local factors that would need to be considered in interpreting the data. For example, mobility patterns in Hong Kong may have been reduced over the past year due to the pro-democracy demonstrations. Third, Citymapper has only published its Mobility Index since the beginning of the pandemic and, while there is now more data available since the time of original writing, we sought to focus on the initial, comparative stages of the epidemic where critical variations in country policy responses took place. Fourth, although the data include cities from across the world, there are some notable gaps, such as mainland China and India. Fifth, as the data are user-generated, there may be selection bias due to the characteristics of those using the app, especially because the app is less widely used for private transportation. More specifically, the sample might over represent those individuals using public transportation compared with those using privately owned cars. Another study using mobile phone data showed how, in New York City, there was a substantially greater reduction in mobility among residents of wealthy areas than poor ones, reflecting the greater ability of the former to work from home [16]. Moreover, given that some measures were introduced at later stages in the pandemic in each country, our models may underestimate the full effect of those measures as more people were already subject to restrictions. More recent research on the effect of countermeasures on transmission of the virus can take advantage of emerging individual-level data to address this issue [17].

Finally, while mobility is a reasonable proxy for contact rates, our study has not attempted to model the subsequent impact of mobility restrictions on incidence or transmission of COVID-19. There is some evidence, for example, that in the case of COVID-19 and other novel corona viruses, school closures may have smaller impacts on transmission than is the case with established influenza virus infections [18]. Future research is needed to operationalise mobility measures as a parameter of contact rates in standard susceptible-exposed-infected-recovered (SEIR) or SEAIR (susceptible, exposed, asymptomatic, infectious, removed) epidemiological models of COVID-19 spread, with findings feeding into models of the impact of non-pharmaceutical interventions, such as that developed by Imperial College [19].

Citymapper is only one source of and, as the pandemic progresses, new sources are coming online, although often not in ways that make it easy to conduct analsyes such as the ones we conducted. Thus, Google has published a series of COVID-19 Community Mobility Reports but the graphs are in pdf format and it is not clear that the data are available [20]. The Citymapper data seem, as far as we can ascertain, to be unique in being in the public domain and covering cities worldwide. This makes it possible to do the analyses we have performed and thus understand the consequences of different policies. This is important given the high social costs of these policies, which will inevitably weigh upon politicians called on to make difficult choices.

### Meaning of the study

Our findings suggest that policies to restrict movement are essential for rapid and dramatic reductions in population mobility. This is evidenced, for example, in the steep decline in mobility in the UK after implementation of restrictions, compared with the more modest previous reductions following government advised but not mandated behaviour change. Our data cover the period of initial implementation of restriction policies; questions remain about whether coercive policies are sustainable over long periods, particularly if measures are perceived as socio-economically inequitable [21].

Our analyses suggest that closure of public transport, workplaces and schools achieved substantial reductions in mobility. However, further work is needed to examine how and if these policies could generate unintended consequences including: undermining health systems and other essential services, due to staff’s loss of transport or childcare; economic hardship arising from loss of earning; and increasing children’s social contact with grandparents [22]. Given these considerations, it is likely that politicians will look to pragmatic policies, such as: reducing but not closing public transport services; workplaces and schools remaining open in the case, respectively, of essential services and the children of key workers [23]; and rapid and generous income maintenance programmes. Lifting too many measures at once without appropriate surveillance and safeguards in place may cause a rapid resurgence of transmission, as is already being seen in some American states. However, monitoring mobility changes can inform continuous assessments of policy impact.

### Future research

Unsurprisingly, there is relatively little other research with which to compare these findings. Exceptions include recent reviews of the effect of school closures on disease transmission, finding that this measure has some but not a great impact [18], reducing social contact among students but not to zero and possibly with unintended consequences for mixing across schools and across generations.

Comparing cities in different regions, it does appear that there have been large reductions in mobility across most of Europe, perhaps to a greater degree than was anticipated by policymakers. There is a somewhat different pattern in Asia, where the focus has been much more on case ascertainment, contact tracing and isolation, making use of the capacity to undertake widespread testing. However, it is notable that Singapore, where the reduction in mobility was least, is now implementing restrictions that it had previously avoided [24].

### Conclusion and policy implications

In a world where large numbers of people carry with them devices with what would, until recently, have been considered impossible amounts of computing power, there are many new opportunities open to epidemiologists which, as in this case, can provide new insights into the impact of policy, providing evidence that can be used for safeguarding health and well-being. Very recent work uses phone data to track changes in mobility, which could ultimately be used to obtain more insights on contact rates [17, 25]. Yet, it is also important to remember that such information can be used for other purposes, raising concerns about privacy, and it will always be necessary to balance the opportunities and the threats of the digital environment [26].

## Supplementary information

**Additional file 1.**

## Data Availability

All data are available on request to the corresponding author, david.stuckler@unibocconi.it
