# Supplementary material for: Using volunteered geographic information to assess mobility in the early phases of the COVID-19 pandemic: a cross-city time series analysis of 41 cities in 22 countries from March 2nd to 26th 2020
Source: Global Health. 2020 Sep 23;16:85. doi: 10.1186/s12992-020-00598-9 (PMC7509494; doi:10.1186/s12992-020-00598-9)
Supplement: Supplementary file 1 — Additional file 1. [file 12992_2020_598_MOESM1_ESM.docx]

Appendix 1. Comparison of Citymapper data with official data on journeys in London

Appendix 2. Changes in the Mobility Index in Italian and British cities


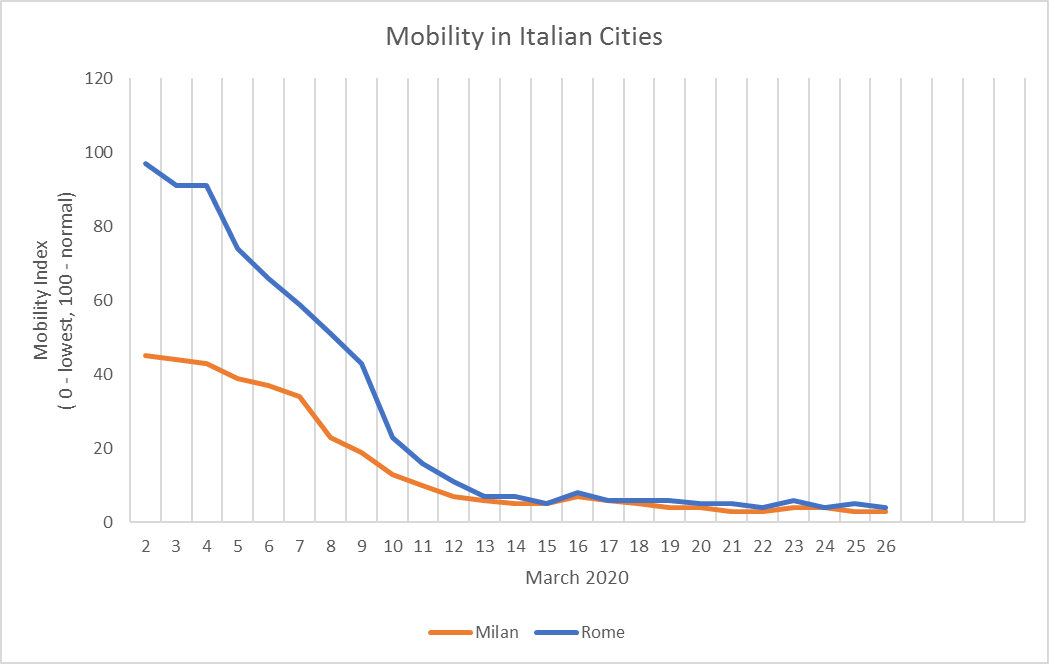


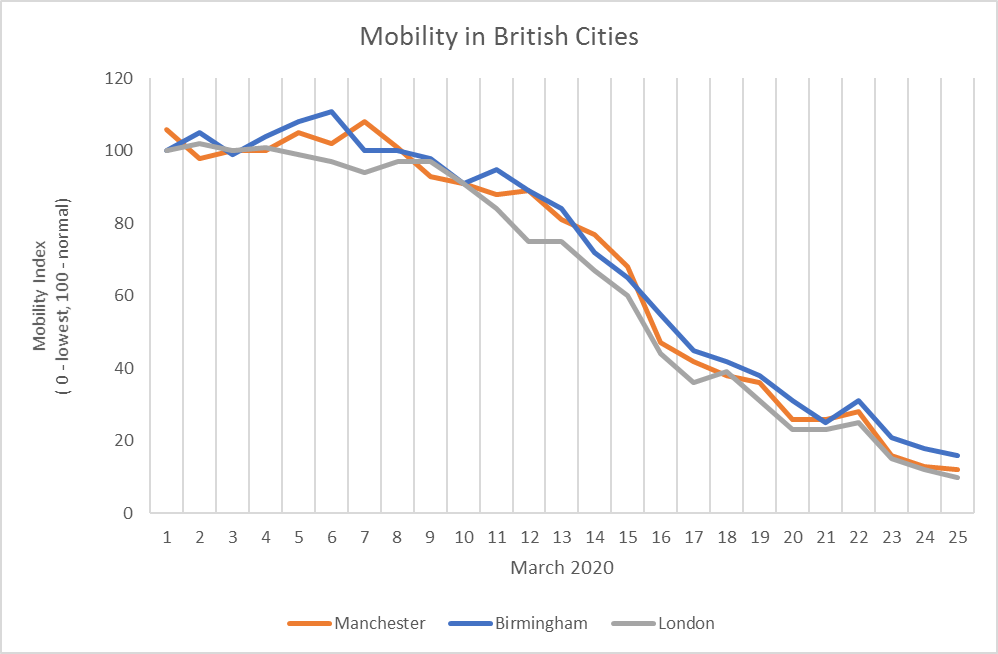


Appendix 3. Unadjusted association of Policy Restrictions with Citymapper’s Mobility Index, 41 cities

|  | Estimated Association of Restriction with |
| --- | --- |
|  | Mobility |
| School closing | -31.9^***^ [-35.0,-28.9] |
| Workplace closing | -35.1^***^  [-40.2,-29.9] |
| Cancel public events | -41.0^***^ [-46.9,-35.1] |
| Close public transport | -57.2^***^ [-80.2,-34.3] |
| Public information campaigns | -44.9^***^ [-52.6,-37.1] |
| Restrictions on internal movement | -37.5^***^ [-43.5,-31.5] |
| International travel controls | -29.2^***^ [-33.6,-24.7] |
| Fiscal measures | -3.4e-10^**^ [-5.4e-10,-1.4e-10] |
| Monetary measures | 52.1^***^ [47.2,57.0] |
| Emergency investment in health care | 8.7e-09***  [7.2e-09,0.000000010] |
|  |  |

Notes: 95% confidence intervals in brackets

^**^ p < 0.01, ^***^ p < 0.001

STROBE checklist of items that should be included in reports of observational studies

|  | Item No | Recommendation |
| --- | --- | --- |
| **Title and abstract** | | |
|  | 1 | (*a*) **time series analysis in title** |
|  |  | (*b*) Trends were evaluated using Citymapper’s mobility index covering 2^nd^ to 25^th^ March 2020, expressed as percentage of typical usage periods from 0% as the lowest and 100% as normal. China and India were not covered. Multivariate fixed effects models were used to estimate the association of policies restricting movement on mobility before and after their introduction. Policy restrictions were assessed using the Oxford COVID-19 Government Response Stringency Index as well as measures coding the timing and degree of school and workplace closures, transport restrictions, and cancellation of mass gatherings.  Setting: 41 cities worldwide  Main outcome measures: Citymapper’s mobility index  Results: Mobility declined sharply in all major cities throughout March. Larger declines were seen in European than Asian cities. The COVID-19 Government Response Stringency Index was strongly and negatively associated with mobility (r = -0.75, p<0.001). After adjusting for time-trends, we observed that implementing a mobility restriction to the recommended level was associated with a decline of mobility of 10.0% for school closures (95% CI: 4.36% to 15.7%), 15.0% for workplace closures (95% CI: 10.2% to 19.8%), 7.09% for cancelling public events (95% CI: 1.98% to 12.2%), 18.0% for closing public transport (95% CI: 6.74% to 29.2%), 13.3% for restricting internal movements (95% CI: 8.85% to 17.8%) and 5.30% for international travel controls (95% CI: 1.69 to 8.90). In contrast, as expected, there was no association between population mobility changes and fiscal or monetary measures or emergency healthcare investment. |
| **Introduction** | | |
| Background/rationale | 2 | Reference to COVID-19 plus discussion of literature on use of mobile phone and related technology in epidemics |
| Objectives | 3 | To examine the feasibility of using Citymapper data to capture changes in mobility and relate them to the timing of pandemic countermeasures. |
| **Methods** | | |
| Study design | 4 | Described in methods section |
| Setting | 5 | We specify period 2nd to 25th March 2020 and present map of locations (Figure 1) |
| Participants | 6 | n/a |
|  |  | n/a |
| Variables | 7 | Outcome is changes in mobility, taken from Citymapper database, interventions are countermeasures from Oxford database – specified in methods |
| Data sources/ measurement | 8* | Described in methods |
| Bias | 9 | In discussion under limitations |
| Study size | 10 | We used all the available data |
| Quantitative variables | 11 | In methods |
| Statistical methods | 12 | (*a*) Describe all statistical methods, including those used to control for confounding: in methods |
|  |  | (*b*) Describe any methods used to examine subgroups and interactions: n/a |
|  |  | (*c*) Explain how missing data were addressed: n/a |
|  |  | (*d*) *Cohort study*?If applicable, explain how loss to follow-up was addressed*Case-control study*?If applicable, explain how matching of cases and controls was addressed*Cross sectional study*?If applicable, describe analytical methods taking account of sampling strategy: n/a |
|  |  | (*e*) Describe any sensitivity analyses: n/a but we do report robustness analyses |
| **Results** | | |
| Participants | 13* | (*a*) Report numbers of individuals at each stage of study?eg numbers potentially eligible, examined for eligibility, confirmed eligible, included in the study, completing follow-up, and analysed: n/a |
|  |  | (*b*) Give reasons for non-participation at each stage: n/a |
|  |  | (*c*) Consider use of a flow diagram: n/a |
| Descriptive data | 14* | (*a*)Give characteristics of study participants (eg demographic, clinical, social) and information on exposures and potential confounders: n/a |
|  |  | (*b*) Indicate number of participants with missing data for each variable of interest: none |
|  |  | (*c*) *Cohort study*?Summarise follow-up time (eg average and total amount): n/a |
| Outcome data | 15* | *Cohort study*?Report numbers of outcome events or summary measures over time: n/a |
|  |  | *Case-control study?*Report numbers in each exposure category, or summary measures of exposure: n/a |
|  |  | *Cross sectional study?*Report numbers of outcome events or summary measures: n/a |
| Main results | 16 | (*a*) Report the numbers of individuals at each stage of the study?eg numbers potentially eligible, examined for eligibility, confirmed eligible, included in the study, completing follow-up, and analysed: n/a |
|  |  | (*b*) Give reasons for non-participation at each stage: n/a |
|  |  | (*c*) Consider use of a flow diagram: n/a |
| Other analyses | 17 | Report other analyses done?eg analyses of subgroups and interactions, and sensitivity analyses: see robustness analysis |
| **Discussion** | | |
| Key results | 18 | Summarise key results with reference to study objectives: In discussion |
| Limitations | 19 | Discuss limitations of the study, taking into account sources of potential bias or imprecision. Discuss both direction and magnitude of any potential bias: In discussion |
| Interpretation | 20 | Give a cautious overall interpretation of results considering objectives, limitations, multiplicity of analyses, results from similar studies, and other relevant evidence: In discussion |
| Generalisability | 21 | Discuss the generalisability (external validity) of the study results: In discussion |
| **Other information** | | |
| Funding | 22 | Give the source of funding and the role of the funders for the present study and, if applicable, for the original study on which the present article is based: Statement included |

Appendix 1. Comparison of Citymapper data with official data on journeys in London

Appendix 2. Changes in the Mobility Index in Italian and British cities


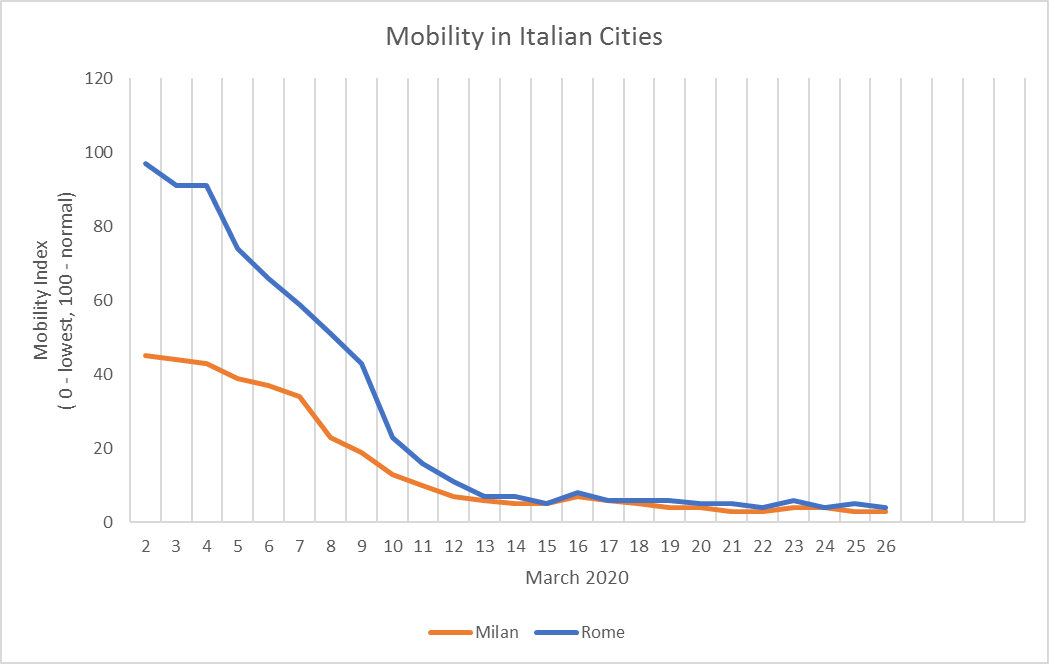


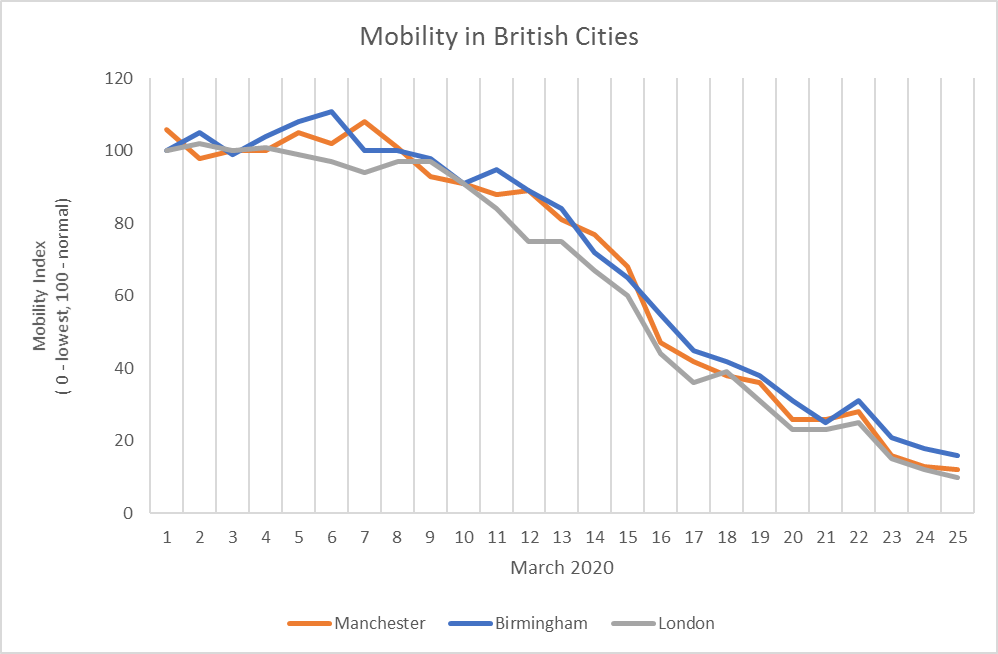


Appendix 3. Unadjusted association of Policy Restrictions with Citymapper’s Mobility Index, 41 cities

|  | Estimated Association of Restriction with |
| --- | --- |
|  | Mobility |
| School closing | -31.9^***^ [-35.0,-28.9] |
| Workplace closing | -35.1^***^  [-40.2,-29.9] |
| Cancel public events | -41.0^***^ [-46.9,-35.1] |
| Close public transport | -57.2^***^ [-80.2,-34.3] |
| Public information campaigns | -44.9^***^ [-52.6,-37.1] |
| Restrictions on internal movement | -37.5^***^ [-43.5,-31.5] |
| International travel controls | -29.2^***^ [-33.6,-24.7] |
| Fiscal measures | -3.4e-10^**^ [-5.4e-10,-1.4e-10] |
| Monetary measures | 52.1^***^ [47.2,57.0] |
| Emergency investment in health care | 8.7e-09***  [7.2e-09,0.000000010] |
|  |  |

Notes: 95% confidence intervals in brackets

^**^ p < 0.01, ^***^ p < 0.001

STROBE checklist of items that should be included in reports of observational studies

|  | Item No | Recommendation |
| --- | --- | --- |
| **Title and abstract** | | |
|  | 1 | (*a*) **time series analysis in title** |
|  |  | (*b*) Trends were evaluated using Citymapper’s mobility index covering 2^nd^ to 25^th^ March 2020, expressed as percentage of typical usage periods from 0% as the lowest and 100% as normal. China and India were not covered. Multivariate fixed effects models were used to estimate the association of policies restricting movement on mobility before and after their introduction. Policy restrictions were assessed using the Oxford COVID-19 Government Response Stringency Index as well as measures coding the timing and degree of school and workplace closures, transport restrictions, and cancellation of mass gatherings.  Setting: 41 cities worldwide  Main outcome measures: Citymapper’s mobility index  Results: Mobility declined sharply in all major cities throughout March. Larger declines were seen in European than Asian cities. The COVID-19 Government Response Stringency Index was strongly and negatively associated with mobility (r = -0.75, p<0.001). After adjusting for time-trends, we observed that implementing a mobility restriction to the recommended level was associated with a decline of mobility of 10.0% for school closures (95% CI: 4.36% to 15.7%), 15.0% for workplace closures (95% CI: 10.2% to 19.8%), 7.09% for cancelling public events (95% CI: 1.98% to 12.2%), 18.0% for closing public transport (95% CI: 6.74% to 29.2%), 13.3% for restricting internal movements (95% CI: 8.85% to 17.8%) and 5.30% for international travel controls (95% CI: 1.69 to 8.90). In contrast, as expected, there was no association between population mobility changes and fiscal or monetary measures or emergency healthcare investment. |
| **Introduction** | | |
| Background/rationale | 2 | Reference to COVID-19 plus discussion of literature on use of mobile phone and related technology in epidemics |
| Objectives | 3 | To examine the feasibility of using Citymapper data to capture changes in mobility and relate them to the timing of pandemic countermeasures. |
| **Methods** | | |
| Study design | 4 | Described in methods section |
| Setting | 5 | We specify period 2nd to 25th March 2020 and present map of locations (Figure 1) |
| Participants | 6 | n/a |
|  |  | n/a |
| Variables | 7 | Outcome is changes in mobility, taken from Citymapper database, interventions are countermeasures from Oxford database – specified in methods |
| Data sources/ measurement | 8* | Described in methods |
| Bias | 9 | In discussion under limitations |
| Study size | 10 | We used all the available data |
| Quantitative variables | 11 | In methods |
| Statistical methods | 12 | (*a*) Describe all statistical methods, including those used to control for confounding: in methods |
|  |  | (*b*) Describe any methods used to examine subgroups and interactions: n/a |
|  |  | (*c*) Explain how missing data were addressed: n/a |
|  |  | (*d*) *Cohort study*?If applicable, explain how loss to follow-up was addressed*Case-control study*?If applicable, explain how matching of cases and controls was addressed*Cross sectional study*?If applicable, describe analytical methods taking account of sampling strategy: n/a |
|  |  | (*e*) Describe any sensitivity analyses: n/a but we do report robustness analyses |
| **Results** | | |
| Participants | 13* | (*a*) Report numbers of individuals at each stage of study?eg numbers potentially eligible, examined for eligibility, confirmed eligible, included in the study, completing follow-up, and analysed: n/a |
|  |  | (*b*) Give reasons for non-participation at each stage: n/a |
|  |  | (*c*) Consider use of a flow diagram: n/a |
| Descriptive data | 14* | (*a*)Give characteristics of study participants (eg demographic, clinical, social) and information on exposures and potential confounders: n/a |
|  |  | (*b*) Indicate number of participants with missing data for each variable of interest: none |
|  |  | (*c*) *Cohort study*?Summarise follow-up time (eg average and total amount): n/a |
| Outcome data | 15* | *Cohort study*?Report numbers of outcome events or summary measures over time: n/a |
|  |  | *Case-control study?*Report numbers in each exposure category, or summary measures of exposure: n/a |
|  |  | *Cross sectional study?*Report numbers of outcome events or summary measures: n/a |
| Main results | 16 | (*a*) Report the numbers of individuals at each stage of the study?eg numbers potentially eligible, examined for eligibility, confirmed eligible, included in the study, completing follow-up, and analysed: n/a |
|  |  | (*b*) Give reasons for non-participation at each stage: n/a |
|  |  | (*c*) Consider use of a flow diagram: n/a |
| Other analyses | 17 | Report other analyses done?eg analyses of subgroups and interactions, and sensitivity analyses: see robustness analysis |
| **Discussion** | | |
| Key results | 18 | Summarise key results with reference to study objectives: In discussion |
| Limitations | 19 | Discuss limitations of the study, taking into account sources of potential bias or imprecision. Discuss both direction and magnitude of any potential bias: In discussion |
| Interpretation | 20 | Give a cautious overall interpretation of results considering objectives, limitations, multiplicity of analyses, results from similar studies, and other relevant evidence: In discussion |
| Generalisability | 21 | Discuss the generalisability (external validity) of the study results: In discussion |
| **Other information** | | |
| Funding | 22 | Give the source of funding and the role of the funders for the present study and, if applicable, for the original study on which the present article is based: Statement included |
